# Infants with Down syndrome and congenital heart disease have altered peri-operative immune responses

**DOI:** 10.1038/s41390-022-02000-3

**Published:** 2022-03-29

**Authors:** Lyudmyla Zakharchenko, Afif EL-Khuffash, Tim Hurley, Lynne Kelly, Ashanti Melo, Maureen Padden, Orla Franklin, Eleanor J. Molloy

**Affiliations:** 1 Paediatric Cardiology, Children’s Health Ireland at Crumlin & Tallaght, Dublin, Ireland; 2 National Children’s Research Centre, Crumlin, Dublin, Ireland; 3 Department of Neonatology, Rotunda Hospital, Dublin, Ireland; 4 Department of Paediatrics, Royal College of Surgeons in Ireland, Dublin, Ireland; 5 Paediatrics, Trinity College, The University of Dublin, Trinity Research in Childhood Centre (TRiCC) & Trinity Translational Medicine Institute (TTMI), Dublin, Ireland; 6 Paediatrics, Coombe Women and Infants University Hospital, Dublin, Ireland; 7 Neonatology, Children’s Health Ireland at Crumlin & Tallaght, Dublin, Ireland

## Abstract

**Background:**

Infants with Down syndrome (DS) have an altered immune response. We aimed to characterise the inflammatory response in infants with DS and congenital heart disease (CHD) peri-operatively in comparison to infants with CHD and a normal chromosomal complement, and to healthy infants pre-operatively.

**Methods:**

Infants with DS/CHD, infants without DS but with CHD (CHD only) and healthy infants were prospectively recruited and serial serum cytokines evaluated peri-operatively using multiplex ELISA: tumour necrosis factor (TNF)-α and TNF-β; interferon (IFN)-γ, interleukin (IL)-1α, IL-2, IL-6, IL-8, IL-18, IL-1β, IL-10, and IL-1ra; vascular endothelial growth factor (VEGF); granulocyte macrophage colony-stimulating factor (GM-CSF); and erythropoietin (EPO).

**Results:**

Ninety-four infants were recruited including age-matched controls (*n* = 10), DS/CHD (*n* = 55), and CHD only (*n* = 29). Children with DS/CHD had significantly lower concentrations of several cytokines (IL-10, IL-6, IL-8, IL-1β, VEGF) in the pre- and post-operatively vs CHD only and controls. EPO and GM-CSF were significantly higher in DS/CHD (*p* value <0.05).

**Conclusions:**

Children with DS/CHD had significantly lower concentrations of several cytokines compared to controls or children with CHD only. EPO and GM-CSF were significantly higher in children with DS/CHD. The assessment of the immune response may be suitable for the predictable clinical outcomes in these children.

**Impact:**

- This study demonstrated that children with Down syndrome (DS) and congenital heart disease (CHD) have significant alterations in pro-inflammatory and anti-inflammatory immune responses peri-operatively.
- These changes may contribute to adverse clinical outcomes, including sepsis, chylothorax, and autoimmunity. They may impact the pathogenesis and outcome post-operatively and long term in this population.
- Children with DS and CHD have significantly lower cytokine concentrations, increased EPO and GM-CSF, and decreased VEGF pre- and post-operatively. Assessing their inflammatory state peri-operatively may facilitate the development of a predictive model that can inform tailored management of these infants using novel therapies including immunomodulation.

## Introduction

Down syndrome (DS) is the most common recognisable chromosomal anomaly of live-born infants worldwide, and it occurs with an incidence of 1:700 in the USA and 1:546 in Ireland, which is the highest incidence of DS in Europe.^1,2^ DS is also the most common syndrome associated with abnormal immune function and immune deficits including both innate and adaptive immunity affected.^3,4^ Altered innate immune function is common and includes altered Toll-like receptor signalling and decreased endotoxin responses.^5,6^ This dysfunction is attributed to a combination of decreased B-lymphocytes, increased CD14 and CD16 pro-inflammatory monocytes, and alterations in the levels of tumour necrosis factor-alpha (TNF-α) and interleukin 6 (IL-6).^7–9^

Cytokines are proteins that facilitate communications and interactions between elements of the immune system.^10^ In the dysregulation of the pro- and anti-inflammatory cytokines released in the setting of infection, a systemic inflammatory response or a compensatory anti-inflammatory response may occur, which potentially can lead to adverse outcomes.^11^ A recent meta-analysis of cytokine profiles in children with DS found increased pro-inflammatory mediators, such as IL-1, TNF-α, and interferon γ (IFN-γ).^12^ EPO plays a key role in the modulation of the response to injury, inflammation, and tissue hypoxia via the inhibition of apoptosis,^13^ while vascular endothelial growth factor (VEGF) plays an important role in regulating angiogenesis, lymphangiogenesis and is associated with tumour growth and chronic inflammation.^7,14^

Children with DS are more susceptible to severe infections, with an increased length of hospital stay due to respiratory infections and more frequent intensive care and ventilator support.^15,16^ Infants with DS have a sixfold increased risk of requiring extracorporeal membrane oxygenation (ECMO) due to respiratory failure when compared to infants without DS and they are at a significantly higher risk of death following ECMO (35 vs 25%).^17^ The mortality rate in critically sick infants and children with DS admitted to intensive care units is very high (40%).^18^ There is increasing recognition of a higher risk for adverse short-term peri-operative outcomes occurring in this population. These include an increase in intensive care and total hospital stay, an increased risk of sepsis, a higher incidence of post-operative chylothorax and increased mortality.^19,20^

Children with DS had a more pronounced immune response when compared with non-DS patients.^21^ The impact of this pro-inflammatory state on short-term outcomes in infants with DS in the peri-operative period is not clearly understood. The assessment of the inflammatory state in the peri-operative period could describe the effect of DS in infants with CHD on complications and morbidities post-operatively and could have a significant impact on their monitoring and management peri-operatively. We aimed to characterise the inflammatory response in children with DS/CHD and children with CHD and a normal chromosomal complement in the peri-operative period.

## Methods

### Study setting and patient population

Ethical approval was granted from the Ethics and Research Board of the Children’s Health Ireland (CHI) at Crumlin (GEN/647/18; 6/04/2018). This was a prospective observational study at The Children’s Heart Centre of CHI at Crumlin between July 2018 and July 2020. The Children’s Heart Centre in CHI at Crumlin is the only Paediatric Cardiac Surgical Unit in Ireland. Five hundred cardiac surgeries (350 bypass cases) are performed annually. Over 20% of children undergoing corrective cardiac surgery have a diagnosis of DS.

The inclusion criteria were infants with a confirmed diagnosis of DS admitted for surgical repair (open heart surgery with patch closure of the lesion) of congenital heart disease (CHD). The lesions included were complete atrioventricular septal defect (cAVSD) and ventricular septal defect (VSD). A group of infants with a normal chromosomal complement and CHD (CHD only) were recruited for comparison and matched as much as practical for the type of the lesion, sex, weight and age of surgery. Their gestational age was >33 weeks and with age prior to surgical intervention between 3 and 7 months. The third group of healthy infants between 3 and 7 months of age with neither DS nor CHD were recruited as controls from outpatient clinics, who attended for routine follow-up of growth or development and who were subsequently normal with a structurally normal heart.

The exclusion criteria for the study were as follows: suspected or definite chromosomal or genetic abnormalities other than DS, cyanotic or obstructive cardiac lesions (including tetralogy of Fallot), major comorbidities (cancer, liver, and kidney diseases, brain abnormalities, or requiring prolonged intensive care support in the neonatal period) and were <33 weeks of gestational age.

### Clinical data collection

Clinical data collected included: gestational age; birth weight; sex; weight and age at the time of the surgery; co-morbidities and complications; mortality; duration of ventilation; nitric oxide uses and duration; inotrope support was captured by using calculation of the vasoactive inotropic score (VIS) as follows: VIS = Dopamine + Dobutamine + (100 × Adrenalin) + (100 × Noradrenalin) + (10 × Milrinone) + (10,000 × Vasopressin)^22^; length of intensive care; and hospital stay. In addition, surgical details such as aortic cross-clamp time (Xclamp) and cardiovascular bypass time (CPB) were also collected.

### Blood sampling

Three blood samples were obtained in surgical cohorts: first—pre-operative, second—post-operative (in 24 h after the surgery) and third—on the day prior to planned discharge from the hospital for biochemical parameters and cytokines. The age-matched control group not undergoing surgery had one sample. Blood samples were timed with routine phlebotomy for other clinical indications and 1–1.5 mL was collected into a Lithium–heparin bottle and centrifuged immediately after collection at 20 °C, 3000 rpm for 5 min. Plasma was frozen at −80 °C for later batch analysis and blood samples were rejected if grossly haemolysed.

### Multiplex cytokine analysis

Pro- and anti-inflammatory cytokines were evaluated to quantify the systemic pro- and anti-inflammatory response in children who were undergoing corrective surgical repair in the peri-operative period, including ILs (IL-2, IL-6, IL-8, IL-18, IL-1β, IL-1α, IL-10, IL-1ra), TNF-α, TNF-β, IFN-γ, VEGF, granulocyte macrophage colony-stimulating factor (GM-CSF), and erythropoietin (EPO). Peripheral blood plasma was transferred to a 96 well MSD plate and cytokines were analysed according to the manufacturer’s instructions (Meso Scale Discovery, Rockville, MD, USA, www.meso-scale.com). The assays employed a sandwich immunoassay format where capture antibodies were coated in a patterned array, on the bottom of the wells of a MULTI-SPOT plate. Assays were readily transferred to the U-PLEX platform with calibration curves showing expected signals, sensitivity precision, and accuracy. Sensitivities were <1 pg/ml for many assays. All assays used the same diluents. Non-specific binding between assays was typically <0.1%. U-plex sample recovery is within the acceptable range (70–130%) with samples diluting linearly from 2- to 16-fold.^23^

### Statistical analysis

Statistical analysis was performed using SPSS Version 26 (www.ibm.com/SPSS Statistic). Data were assessed for normal distribution using Kolmogorov–Smirnov test, with a *p* value <0.05 interpreted as evidence against the presumption of a normal distribution. When data were non-normally distributed, a log transformation was applied and the data were re-assessed. Greenhouse–Geisser correction required and applied to tests of within-subjects effects were appropriate. A two-way analysis of variance was used to compare serial cytokine results peri-operatively. Statistical significance was achieved with a *p* value <0.05.

## Results

In total, 94 infants were recruited (*n* = 196 samples): DS and CHD (DS/CHD including cAVSD and VSD; *n* = 55), non-DS and CHD (CHD only; *n* = 29) and were matched for cardiac lesion, age, weight (pre-intervention), and sex. Healthy controls (*n* = 10) were recruited for comparison with surgical groups pre-operatively (Table 1). Infants in the DS/CHD group had a significantly lower gestational age at birth but had no differences in the birth weight or in the weight and age at the time of surgery when compared to the CHD group (Table 1). In comparison, infants with DS/CHD to healthy infants had lower birth weight and weight prior to intervention (Table 1). None of the infants was intubated or was on any inotrope support prior to the surgical intervention. Also, none of them had any signs of infections or had an abnormal CRP prior to surgery.

**Table 1:** Demographics of healthy infants compared to children having surgery with DS/CHD and CHD.

	Controls *n* = 10	DS/CHD *n* = 55	CHD *n* = 29	*p	**p	***p
Gestation at birth (weeks)	39.4 [37.8–40.2]	37.8 [36.3–38.7]	39.0 [37.0–40.0]	**0.01**	0.05	0.48
Birth weight (kg)	3.15 [3.08–3.61]	2.92 [2.57–3.26]	3.17 [2.64–3.60]	0.22	**0.02**	0.15
Female	5 (50)	31 (56)	17 (59)	1.00	0.92	0.81
Age at surgery (months)	6 [3–7]	5 [4–6]	5 [4–6]	0.19	0.35	0.16
Weight at surgery (kg)	6.7 [5.6–7.6]	5.53 [5.06–6.08]	5.33 [4.86–6.28]	0.68	**0.03**	0.2

Values are presented as median [interquartile range (IQR)] or count (%) as appropriate.

*p* Value of <0.05 was considered significant (*p value compared DS/CHD to CHD; **p value compared DS/CHD to Controls; ***p value compared CHD to Controls).

Statistically significant *p*-values are in bold.

Infants in the DS/CHD group had a longer cross-clamp time, a longer cardiopulmonary bypass time, greater inotropic support with a significantly higher vasoactive inotropic score (12 [9–16] vs 5 [5–8] in CHD cohort, *p* < 0.01) (Table 2). The duration of inotropic support was also significantly longer in infants with DS/CHD (2 [1–4] vs 1 [1] days, p < 0.01) (Table 2). Infants in the DS/CHD group were more likely to require treatment with nitric oxide and had a significantly longer duration of ventilation (Table 2). The total duration of PICU stay post-operatively and the total length of hospital stay was significantly longer in infants with DS/CHD (Table 2). Interestingly, chylothorax was seen exclusively in the DS/CHD population (Table 2). In this study mainly infants with DS/CHD had respiratory infections in the post-operative period 16 vs 0% in infants with CHD only. Septicaemia was seen in 11% in infants with DS/CHD group and only 3% vs in CHD only, with no significant difference between the groups. The DS/CHD group developed a significantly higher incidence of heart block post-operatively (Table 2). There was no significant difference in mortality between the two groups, in those who had severe sepsis post-operatively and did not survive despite the escalation of care (Table 2).

**Table 2:** Characteristics and the clinical outcomes in the two surgical groups.

	DS/CHD *n* = 55	CHD *n* = 29	*p*
Intra and post-op characteristics			
Cross-clamp time (minutes)	76 [63–105]	53 [40–62]	<0.01
Cardiopulmonary bypass time (min)	107 [92–145]	80 [67–95]	<0.01
Vasoactive-inotropic score (first 24 h post-op)	12 [9–16]	5 [5–8]	<0.01
Heart block	19 (35%)	1 (3%)	<0.01
Respiratory infection	9 (16%)	0	0.02
Septicaemia	6 (11%)	1 (3%)	0.41
Chylothorax	24 (44%)	0	<0.01
Nitric oxide	10 (18%)	1 (3%)	0.09
Duration of inotropes (days)	2 [1–4]	1 [1]	<0.01
Duration of ventilation (days)	3 [2–5]	1 [1]	<0.01
Duration of PICU stay (days)	5 [3–7]	2 [2–6]	<0.01
Length of hospital stay (days)	12 [9–17]	7 [6–10]	<0.01
Mortality	1 (2%)	1 (3%)	1.00

Values are presented as median [interquartile range (IQR)] or count (%) as appropriate.

*PICU* Paediatric Intensive Care Unit.

*p* Value of <0.05 was considered significant.

### IL-2 and TNF-α

**Fig. 1:**
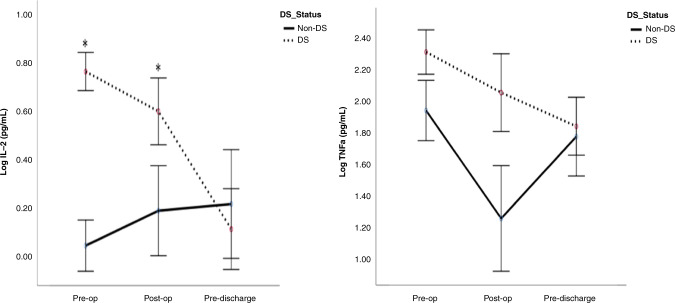
Interleukin 2 (IL-2) and tumour necrosis factor-α (TNF- α) in children with DS/CHD vs CHD undergoing cardiac surgery. Cytokines were evaluated by multiplex ELISA and values are presented as means (circles), *p < 0.05 comparing children with DS and CHD (black dots) to children without DS with CHD (CHD only; black line). Pre-op pre-operatively, Post-op post-operatively, DS Down syndrome.

Infants with DS/CHD had statistically significantly higher concentrations of IL-2 and TNF-α in the pre-operative and post-operative periods when compared to CHD infants (Fig. 1). However, there was no significant difference in the pre-discharge period in those concentrations between infants with DS/CDH vs non-CDH (Fig. 1). There was also no difference in comparison of both groups to controls pre-operatively (Table 3).

**Table 3:** Cytokines (measurements in pg/ml) in children DS/CHD and CHD in comparison to controls in the pre-operative period.

Cytokine	Control	Pre-op		Post-op		Pre-discharge		*p value	**p value
		DS/CHD	CHD	DS/CHD	CHD	DS/CHD	CHD		
Epo	103 (84.4–379.4)	171.7 (59.4–16551)	114.8 (29.3–365.6)	931.6 (52.3–25496)	524.2 (84.8–3648)	131.3 (44.7–824.1)	117.3 (33.5–3614.7)	0.04	0.43
VEGF	140.9 (17–443.4)	0.001 (0.001–5.5)	143.3 (63.9–1517)	0.001 (0.001–0.001)	114 (51.9–595.2)	488.8 (97.3–2704.8)	269.8 (64.7–2534.8)	0.002	0.38
GMCSF	1.12 (0.2–2.5)	2.6 (1.1–13.8)	0.8 (0.2–22.4)	2.1 (1.1–4.3)	0.5 (0.3–82.8)	1.6 (0.8–66.3)	1.8 (1.0–82.9)	0.0006	0.09
IL-1a	0.001 (0.001–11.2)	0.001 (0.001–36.6)	1.0 (0.001–586.8)	0.001 (0.001–30.6)	0.001 (0.001–132.3)	0.001 (0.001–26.4)	0.001 (0.001–297.3)	0.36	0.14
IL-1b	0.10 (0.1–0.7)	0.001 (0.001–0.001)	0.01 (0.1–1.0)	0.001 (0.001–0.2)	0.4 (0.2–24)	0.3 (0–0.9)	0.3 (0.1–4.3)	0.01	0.39
IL-1ra	568.6 (355.5–827.8)	618.8 (236.8–2301)	544.8 (255–4876)	4630 (624.3–10,518.1)	3197 (627.3–11,196)	855.1 (355.5–2284.1)	851.6 (263.5–3449.4)	0.03	0.06
IL-2	0.001 (0.001–0.1)	1.2 (0.001–2.5)	0.001 (0.001–1.2)	0.9 (0.001–3.3)	0.001 (0.001–8.8)	0.001 (0.001–8.0)	0.001 (0.001–6.4)	5.6	0.13
IL-6	1.0 (0.1–13.7)	0.001 (0.001–0.8)	1.1 (0.3–35.5)	0.001 (0.001–0.8)	74.6 (1.3–546.4)	11.3 (2.4–43.4)	9.6 (0.8–32.5)	0.07	0.15
IL-8	15.4 (0.03–38.8)	0.001 (0.001–0.3)	13.2 (3.5–51.7)	0.001 (0.001–0.6)	36.6 (0.6–155.6)	29.9 (4.7–153.6)	35.5 (11.1–122)	0.001	0.28
IL-10	2.5 (0.7–3.8)	0.001 (0.001–0.2)	1.7 (0.4–30.3)	0.001 (0.001–0.001)	3.1 (0.2–40.7)	2.7 (0.6–21.2)	1.6 (0.7–38.3)	0.0003	0.18
IL-18	1218 (614–1684)	798 (246.9–2419)	734.4 (95.9–3064)	919.3 (279–1852)	837.3 (383.6–1992.8)	746.2 (159.6–1672.5)	573.5 (173.2–1794.1)	0.67	0.13
TNF-a	8.6 (6.4–13.6)	10.7 (2.2–16)	7.2 (3.2–35.3)	7.9 (5.0–14.5)	3.9 (2.1–44)	5.4 (2.1–27.1)	5.5 (4.5–17.1)	0.07	0.47
TNF-b	0.6 (0.1–1.8)	0.2 (0.02–0.9)	0.4 (0.1–3.6)	0.04 (0.001–1.0)	0.01 (0.001–0.7)	0.4 (0.001–2.5)	0.5 (0.3–2.3)	0.01	0.16
IFN-g	142 (4.5–537.7)	0.001 (0.001–17.6)	67.4 (22.2–2196)	0.001 (0.001–2.6)	29.8 (18.8–531.8)	54.7 (11.1–490.7)	88.1 (26–267.7)	0.01	0.48

Values are presented as median [interquartile range (IQR)] or count (%) as appropriate.

*p* Value of <0.05 was considered significant (*p value compared DS/CHD to Controls; **p value compared CHD to Controls). Value 0.00 given as ×10^−3^.

### IL-6, IL-1β, IL-8, IL-1α, IL-10, and IFN-γ

**Fig. 2:**
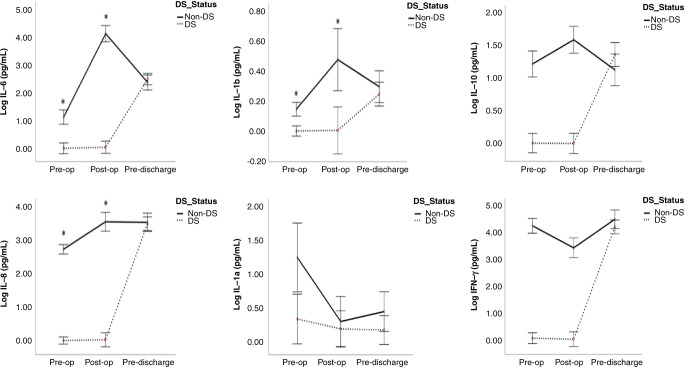
Interleukin-6 (IL-6), interleukin-1β (IL-1β), interleukin-8 (IL-8), interleukin-1α (IL-1α), interleukin-10 (IL-10), and interferon-γ (IFN-γ) in children with DS/CHD vs CHD undergoing cardiac surgery. Cytokines were evaluated by multiplex ELISA and values are presented as means (circles), *p < 0.05 comparing children with DS and CHD (black dots) to children without DS with CHD (black line). Pre-op pre-operatively, Post-op post-operatively, DS Down syndrome.

Children with DS/CHD had significantly lower concentrations of IL-6, IL-1β, IL-8, IL-10, and IFN-γ pre- and post-operatively when compared to children with CHD (Fig. 2). While the concentration of IL-1α was significantly lower in infants with DS/CHD pre-operatively in comparison to CHD only infants with no change in post-operative and pre-discharge periods between them (Fig. 2). Only DS/CHD infants demonstrated significantly lower concentrations of IL-8, IL-1β, IL-10 and IFN-γ pre-operatively when compared to controls (Table 3). Both surgical groups demonstrated a nearly similar concentration of IL-1α pre-operatively in comparison to controls (Table 3).

### IL-18, IL-1ra, and TNF-β

**Fig. 3:**
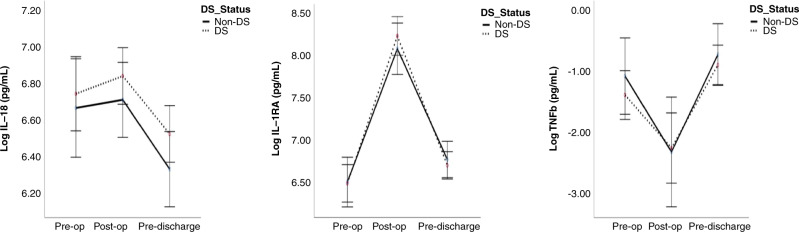
Interleukin-18 (IL-18), interleukin-1ra (IL-1ra), and tumour necrosis factor-β (TNF- β) in children with DS/CHD vs CHD undergoing cardiac surgery. Cytokines were evaluated by multiplex ELISA and values are presented as means (circles), *p < 0.05 comparing children with DS and CHD (black dots) to children without DS with CHD (black line). Pre-op pre-operatively, Post-op post-operatively, DS Down syndrome.

IL-18, IL-1ra, and TNF-β did not significantly differ peri-operatively between DS/CHD and CHD only groups (Fig. 3). There was no difference in IL-18 between the groups and controls pre-operatively (Table 3). Infants with DS/CHD had significantly lower TNF-β and significantly higher IL-1ra concentrations pre-operatively in comparison to controls (Table 3).

### Multifunctional cytokines

**Fig. 4:**
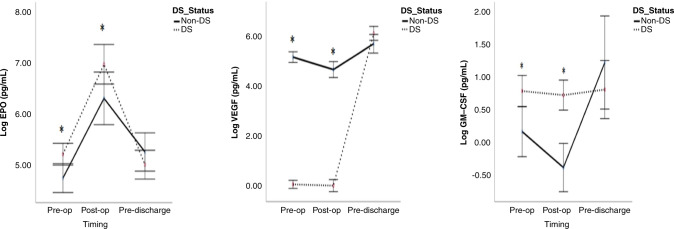
Erythropoietin (EPO) and vascular endothelial growth factor (VEGF), granulocyte macrophage colony-stimulating factor (GM-CSF) in children with DS/CHD vs CHD undergoing cardiac surgery. Cytokines were evaluated by multiplex ELISA and values are presented as means (circles), *p < 0.05 comparing children with DS and CHD (black dots) to children without DS with CHD (black line). Pre-op pre-operatively, Post-op post-operatively, DS Down syndrome.

In infants with DS/CHD, the EPO concentration was significantly higher and VEGF concentration was significantly lower over the pre- and post-operative periods compared to the CHD only infants and was similar in both at discharge. In controls, there were similar results with significantly increased EPO and decreased VEGF vs DS/CDH (Fig. 4 and Table 3).

GM-CSF was significantly higher in the DS/CHD population during the pre- and post-operative periods than CHD only and controls. GM-CSF was similar between DS/CDH and CDH only at discharge (Fig. 4 and Table 3).

## Discussion

Children with DS are an important sub-group in the paediatric cardiac surgery population. The incidence of DS has traditionally been higher in Ireland than in other European countries due to restricted access to termination of pregnancy.^24^ In our surgical programme infants with DS represent over 20% of all children undergoing surgical repair of CHD. The growth was optimised by the multidisciplinary team (MDT) group and pre-operative weight same across the populations studied. As such, previously described problems such as lower operative weight in the DS/CHD population was not present in our cohort.^25^

In this prospective observational study, we demonstrated that infants with DS and CHD undergoing corrective surgery have an increased rate of adverse post-operative outcomes when compared with infants with CHD only. The increased structural cardiovascular complexity was the most likely contributing factor to the observed increase in both the cardiopulmonary bypass and cross-clamp times in infants with DS/CHD. Increased operative complexity with an increased prevalence of inlet VSDs and left AV valve pathology was also likely a factor in the increased risk of complete heart block requiring pacing in the post-operative period in infants with DS/CHD, as described in the previous studies.^25,26^

Interestingly, in our study chylothorax was seen exclusively in the DS/CHD group post-operatively, which could be possibly secondary to the lymphatic malformations such as congenital lymphangiectasia. DS related lymphangiectasia is a well-recognised complication of this chromosomal abnormality.^8,9,27^ This resulted in an increased duration of pleural drain placement post-operatively in the infants with DS/CHD infants and was a further contributor to the increased duration of total hospital stay.

Infants and children with DS exhibit distinct abnormalities in cells of the innate immune system favouring a pro-inflammatory state.^8,9,27^ Our study did not identify a significantly increased incidence of post-operative septicaemia, but children with DS/CHD had a higher incidence of respiratory infections.

While increased case complexity and DS/CHD related co-morbidities had a significant impact on peri-operative morbidity, duration of PICU stay and total length of hospital stay in the infants with DS/CHD, there was no increase in mortality in this group and none of them required ECMO. This finding is welcome highlighting the potential to ultimately achieve comparable post-operative outcomes in the infants with DS/CHD while acknowledging the increased dependence of this group in the early post-operative period.

In this study, we have clearly demonstrated the difference in the inflammatory response between the two surgical groups in the peri-operative period and may provide an explanation for the significantly higher incidences of chylothorax and respiratory infections in infants with DS/CHD population. Children with DS/CHD had significantly lower concentrations of several cytokines (IL-10, IL-6, IL-8, IL-1β, and VEGF) compared to controls or children with CHD only. In contrast, EPO and GM-CSF were significantly higher in children with DS/CHD. Children in the CHD group had no significant difference in any of the cytokines pre-operatively compared to controls.

Children with DS/CHD had significantly higher levels of IL-2 and TNF-α pre- and post-operatively compared to infants with CHD only and were not significantly different to controls. IL-2 enhances the production of TNF-α^28,29^ which could explain the increased TNF-α as secondary to the stimulation of IL-2.^30^ However, IL-6, IL-8, IL-1β, and IFN-γ in the DS/CHD group were decreased and remained similar pre- and post-operatively, although their concentration increased pre-discharge. Only IL-1α did not change and remained decreased. Huggard et al. found higher levels of IL-6, IL-1ra, IL-1β, and IL-8 in children with DS/CHD requiring surgery vs children with DS not requiring surgery although this difference disappeared after 6 weeks post-operatively.^23^

Anti-inflammatory cytokines are vital to promote homoeostasis in an inflammatory response. IL-10 is an anti-inflammatory cytokine that reduces inflammation by inhibiting the production of pro-inflammatory cytokines such as IL-6 and TNF-α.^31^ We have shown a significantly lower IL-10 level in children with DS/CHD pre- and post-operatively which reaches similar levels to children with CHD only at discharge. Lei et al. also demonstrated increased levels of TNF- α and decreased levels of IL-10 in adults following cardiopulmonary bypass in the presence of PH.^32^ In rats IL-10 administration reduced PH and improved survival significantly.^33^ Nategi et al. found lower serum levels of IL-10 in children with DS and a reciprocal increased level of pro-inflammatory cytokines (TNF-α and IFN-γ).^34^ We have also confirmed this increased level of the pro-inflammatory cytokine TNF-α, but IFN-γ was significantly lower level pre- and post-operatively and only increased pre-discharge. Huggard et al. demonstrated increased IL-10 and IL-1ra in children with DS. IL-1ra is an inhibitor of the pro-inflammatory effect of IL-1β.^35^ IL-1ra is sensitised in many tissues in response to local inflammation.^36^ In our study in comparison to the control group only IL-1ra was significantly higher in children with DS/CHD pre-operatively. However, the infants with DS/CHD in the Huggard et al. study were >6 weeks post-operative when sampled.^23^ Alterations in IL-10 peri-operatively in children with DS/CHD may reflect higher local inflammation secondary to the larger defect such as cAVSD, which requires a significantly longer cardiopulmonary bypass time and cross-clamp time.

GM-CSF was significantly elevated throughout the study period in the DS/CHD population which corroborates other research in children with DS.^15^ GM-CSF also plays an important role in chronic and local tissue inflammation and promotes leucocyte maturation, activation and movement from the bone marrow with an associated neutrophilia.^37^ Other most recent studies reported that GM-CSF is also associated with neurological dysfunction in neonates with moderate and severe neonatal encephalopathy, which demonstrated elevation of GM-CSF at days of life 6 and 7 correlated negatively with composite cognitive, language and motor Bayley-3 scores at 2 years.^38^ Also, previously it has been reported that term infants with late diagnosis of cerebral palsy demonstrated a significantly higher concentration of GM-CSF on the newborn screening test in comparison to a matched control.^39^

EPO and VEGF are important in the response to hypoxia as well as promoting new blood vessels and repair of normal tissue.^40–43^ VEGF plays an important role in regulating angiogenesis and lymphangiogenesis.^7,14^ In our study, children with DS/CHD had higher EPO and lower VEGF levels at the pre- and post-operative periods than children with CHD only and pre-operatively than controls. In contrast to EPO, VEGF was significantly lower peri-operatively in the DS/CHD group but increased prior to discharge. These changes, increased GM-CSF and EPO but decreased VEGF, were found in a previous study also in children with DS aged between 1 and 15 years.^34^ A most recent study demonstrated that children with DS and CHD requiring surgery exhibit significantly greater levels of all Epo, VEGF, and GM-CSF than in children with DS/CHD not requiring surgery and with DS who had a normal heart.^23^ However, the infants with DS/CHD in Huggard’s study were >6 weeks post-operative when sampled this may account for the differences in findings.

## Limitations of the study

There are some limitations to this study and the results should be interpreted with caution. There is a relatively smaller number of infants in the control group as they were healthy and as such had low presentation rates to the hospital. Despite the small number of infants in the control group, their baseline cytokines did not differ within the group. The inclusion of this group also allowed standardisation of the measurement techniques used and results obtained with published standard values for normal children.

## Conclusion

This study outlines the additional peri-operative morbidity in infants with DS and CHD when compared to matched infants with CHD only. This data provides a more robust explanation of the difference between these groups following surgical interventions allowing clinicians to tailor pharmacological therapies this may allow for future management strategies tailored to the DS population, can inform post-operative PICU care and recognising the increased morbidity and bed requirements for the DS /CHD group Also, this study demonstrated that children with DS and CHD have significant alterations in pro-inflammatory and anti-inflammatory immune responses as well as increased EPO, decreased VEGF and increased GM-CSF peri-operatively vs children with CHD who have a normal chromosomal complement. The systemic and local inflammation secondary to the larger defect AVSD that required significantly longer surgical intervention was more likely reflected in a lower concentration of IL-10 as an anti-inflammatory marker in the infants with DS group. Increased GM-CSF and EPO and decreased VEGF indeed may have contributed to the higher incidence of chylothorax and longer inotropic support in the DS/CHD population.

These changes in inflammatory responses in children with DS peri-operatively may contribute to the varied clinical outcomes including sepsis and chylothorax, and over time in autoimmunity. In addition, they may impact the pathogenesis and outcome post-operatively and long term in this population. Characterising the inflammatory response in the peri-operative period can pave the way for a tailored management of those infants using novel therapies including immunomodulation. In addition, the output of this project could have a significant impact on the monitoring of infants with DS during the peri-operative period and as such, a robust plan for dissemination and knowledge exchange will be developed. Correlation of cytokines and myocardial function is warranted in the future to develop biomarkers of myocardial function. In addition, monitoring immune function may allow individualised therapies with immunomodulation.
